# Synthesis and Thermoelectric Characterization of Lead Telluride Hollow Nanofibers

**DOI:** 10.3389/fchem.2018.00436

**Published:** 2018-09-24

**Authors:** Miluo Zhang, Su-Dong Park, Jiwon Kim, Michael Nalbandian, Seil Kim, Yongho Choa, Jaehong Lim, Nosang V. Myung

**Affiliations:** ^1^Department of Chemical and Environmental Engineering and UC KIMS Center for Innovation Materials for Energy and Environment, University of California, Riverside, Riverside, CA, United States; ^2^Advanced Materials and Application Research Division, Korea Electrotechnology Research Institute, Changwon, South Korea; ^3^Department of Electrochemistry, Korea Institute of Materials Science, Changwon, South Korea; ^4^Department of Civil Engineering and Construction Management, California Baptist University, Riverside, CA, United States; ^5^Department of Materials Science and Chemical Engineering, Hanyang University, Ansan, South Korea

**Keywords:** lead telluride, electrospinning, galvanic displacement reaction, thermoelectrics, hollow nanofiber, energy barrier height

## Abstract

Lead telluride (PbTe) nanofibers were fabricated by galvanic displacement of electrospun cobalt nanofibers where their composition and morphology were altered by adjusting the electrolyte composition and diameter of sacrificial cobalt nanofibers. By employing Co instead of Ni as the sacrificial material, residue-free PbTe nanofibers were synthesized. The Pb content of the PbTe nanofibers was slightly affected by the Pb^2+^ concentration in the electrolyte, while the average outer diameter increased with Pb^2+^ concentration. The surface morphology of PbTe nanofibers was strongly dependent on the diameter of sacrificial nanofibers where it altered from smooth to rough surface as the Pb^2+^ concentration increased. Some of thermoelectric properties [i.e., thermopower (S) and electrical conductivity(σ)] were systematically measured as a function of temperature. Energy barrier height (E_b_) was found to be one of the key factors affecting the thermoelectric properties–that is, higher energy barrier heights increased the Seebeck coefficient, but lowered the electrical conductivity.

## Introduction

The restriction of non-renewable resources along with the threat of environmental and ecological degradation is a key driver for improving energy generation and efficiency. Various renewable energy technologies including solar cells (Oregan and Gratzel, 1991; Miles et al., 2007), biomasses (Huber et al., 2006), fuel cells (Aricò et al., 2005), and thermoelectrics (Snyder and Toberer, 2008) are considered to achieve the goal. Solid-state thermoelectric generators convert waste thermal energy into useful electric energy to improve the efficient of system. They have many advantages such as long operating time without maintenance, easy scalability, and zero-emission (Pichanusakorn and Bandaru, 2010). The conversion efficiency of the thermoelectric device can be described by the thermoelectric figure-of-merit (*ZT* = *S*^2^σ*T*/κ), which consists of thermopower (Seebeck Coefficient, S), electrical conductivity (σ), and thermal conductivity (κ), and absolute temperature (T). In order to maximize the ZT, there parameters must be independently optimized. However, the interdependence of S, σ, and κ make difficult to further enhance ZT (Szczech et al., 2011). One-dimensional (1-D) nanostructures can offer several advantages in enhancing ZT over bulk materials, such as increasing the power factor, (*S*^2^σ), by means of quantum confinement and/or the energy filtering effect or reducing lattice thermal conductivity (κ_L_) by enhanced phonon scattering (Hochbaum et al., 2008; Chen et al., 2010). Furthermore, both quantum confinement and surface scattering effects are expected to display an added degree of control and enhancement within tubular/hollow nanostructures (*vs*. solid nanowires and nanobelts) (Chen et al., 2010; Zhou et al., 2010, 2014), as wall thickness and fiber diameter can be controlled independently. Thus, tubular nanostructures offer the possibility of decoupling S^2^σ and κ, which allows for independent control over the S^2^σ/ κ ratio, thereby increasing the overall thermoelectric performance (Chen et al., 2010; Zhou et al., 2010, 2014).

Lead telluride (PbTe) is a V-VI semiconductor with a narrow band-gap energy of 0.31 eV at room temperature with a rock-salt crystal structure. By adjusting the composition, PbTe can be either an n- or p-type semiconductor. For example, Te-rich PbTe results in p-type semiconductor whereas Pb-rich PbTe results in n-type semiconductor (Dughaish, 2002). The commercially available PbTe-based thermoelectric devices show ZT of ~0.8 around 600 K, which makes them suitable for the middle-high temperature range. Additionally, the enhancement of the thermoelectric properties of PbTe has already been realized by band-gap engineering. Enhancement in the thermoelectric efficiency of PbTe was also achieved by doping the material with potassium (K) or sodium (Na) (Androulakis et al., 2010). Furthermore, improvement of ZT was achieved via nanoengineering of the Se alloyed PbTe quantum dot superlattice (Harman et al., 2002). The enhancement of ZT (~1.6 at 300 K and ~3.5 at 570 K) in this system is caused by reduction in κ_L_ meanwhile an increase in (*S*^2^σ) (Venkatasubramanian, 2000; Harman et al., 2002, 2005).

Various methods including chemical deposition (Lokhande, 1991; Tai et al., 2008), stress-induced method (Dedi et al., 2013), electrodeposition (Xiao et al., 2006, 2007a; Jung et al., 2011; Yang et al., 2011), galvanic displacement reaction (Chang et al., 2014), CVD (Fardy et al., 2007) have been used to fabricate PbTe nanostructures. The thermoelectric properties of these nanostructures are normally characterized in the form of nanowire arrays or highly-packed nanowire films (pellets). This is due to the difficulties in maintaining the single nanowire's structural and chemical composition during the lithographic contacting (Tai et al., 2008; Yang et al., 2011; Dedi et al., 2013). For example, p-type PbTe nanowires were fabricated using hydrothermal method. The sample were then hot pressed into a pellet for measuring thermoelectric properties, yielding the highest Seebeck coefficient of 628 μV/K (Tai et al., 2008). N-type PbTe nanoribbon arrays with a diameter of 60 nm were synthesized by lithographically patterned nanowire electrodeposition (LPNE), which showed S of −445 μV/K and σ of 0.63 S/cm, respectively (Yang et al., 2011).

Electrospinning is a technique that can produce ultra-long nanofibers by continuously stretching and whipping viscoelastic jets in a high electric field. Various nanofibers of polymer (Xiao et al., 2007a), metal (Xiao et al., 2006), and metal oxide (Yang et al., 2011) materials have been fabricated by electrospinning with controllable morphology, diameter, composition, and orientation. However, limited works are reported on the synthesis of metal chalcongenide nanofibers because of difficulty to prepare solutions. Therefore, synthesis of a few millimeter chalcogenide nanofibers has been realized by combining electrospinning with an additional process called the galvanic displacement reaction (Xiao et al., 2007b; Chang et al., 2010a,b, 2014; Hangarter et al., 2010; Jung et al., 2010, 2012; Park et al., 2010, 2013; Rheem et al., 2010; Suh et al., 2012, 2013; Elazem et al., 2013; Jeong et al., 2013; Liu et al., 2013; Wu et al., 2014, 2015; Zhang et al., 2014), by which the electrospun nanofibers can be converted to desired hollow metal chalcogenides spontaneously. Several hollow nanofibers of chalcogens and metal chalcogenides [e.g., Te (Lee et al., 2011; Jeong et al., 2013), Ag_2_Te (Park et al., 2015; Zhang et al., 2015), and Pb_x_Se_y_Ni_z_ (Zhang et al., 2014)] have been successfully synthesized by this method in our group.

In this paper, hollow PbTe nanofibers with controlled dimension and morphology were synthesized for the first time. Electrospinning was exploited to fabricate sacrificial cobalt nanofibers. Various dimensions and morphologies of the PbTe hollow nanofibers were synthesized by tuning the electrolyte concentrations in the galvanic displacement reactions. Additionally, thermoelectric properties were characterized and correlated to their materials properties.

## Materials and methods

### Electrospinning of co nanofibers

The procedure of electrospinning of cobalt nanofibers is based on previous reported nickel nanofibers (Park et al., 2013). Citric acid (C_6_H_8_O_7_, anhydrous, enzyme grade, Fisher Chemical) and cobalt acetate [Co(C_4_H_6_O_4_). 4H_2_O, 98%, Sigma-Aldrich] at a molar ratio of 1 were dissolved in 6.3 g of water followed by mixing with 3.34 g of anhydrous ethanol solution (Fisher Scientific, PA) containing 5.2 wt. % of polyvinylpyrrolidone (PVP). MW of PVP was 1,300,000 g/mol. The concentration of the precursor solution was varied to electrospin the Co sacrificial nanofibers with two different diameters. The concentration of cobalt acetate was chosen to be 1.0 M (solution 1) and 1.6 M (solution 2) based on the spinnability of the solution and the solubility of the salt. A 0.25 mm inner diameter metallic needle was used as the spinneret. The applied voltage and the distance between spinneret and collector (i.e., 3 × 3 cm Si/SiO_2_ wafer) were fixed at 10 kV and 10 cm, respectively. The flow rate was fixed at 0.5 mL/ h using a peristaltic pump. The electrospinning time was fixed at approximately 15 min to keep the thickness of collected nanofiber mats consistent. The temperature and relative humidity were 40 ± 2°C and 8 ± 1%, respectively. The collected nanofibers were first aged at 60°C in air overnight and then calcined at 500°C in pure H_2_ for 5 h to obtain Co nanofibers.

### Synthesis of PbTe nanofiber mats by galvanic displacement

The galvanic displacement of cobalt to PbTe nanofiber mat was conducted at room temperature for 30 min by dipping a freestanding Co nanofiber mat of the desired amount into 10 ml solution. The solution consisted of X mM lead nitrate (Pb(NO3)2, Fisher Chemical), 0.1 mM tellurium oxide (TeO_2_, 99+%, Acros Organic) and 0.1 M nitric acid (HNO_3_, Certified ACS Plus, Fisher Chemical) The pH of solution was controlled to be 2 by adding nitric acid (HNO_3_, Certified ACS Plus, Fisher Chemical). The effects of Pb^+2^ concentration on the morphology and dimension of the nanofiber were conducted by altering the Pb^2+^concentrations from 10 to 100 mM. After galvanic displacement, the mats were rinsed with de-ionized water five times follow by air dried.

### Solution and material characterization

Various solution properties including electrical conductivity, solution viscosity, and surface tension were measured with Accumet AB-200 benchtop electrical conductivity meter, Brookfield DV-I Prime viscometer, and Interfacial tensiometer (CSC-Denouy 70545), respectively.

Transmission electron microscopy (TEM), selected area electron diffraction (SAED), field emission-scanning electron microscopy (FE-SEM, FEG-Philips XL30), energy-dispersive X-ray spectroscopy (EDS), and X-ray diffraction (XRD, D8 Advance Diffractometer, Bruker) were used to characterize morphologies, compositions, crystal structures and crystallinity of the nanofiber mats.

### Electrical and thermopower characterizations

Single fiber-based devices were fabricated by a standard photolithography process with a gap size of 3 μm between two Au pads. Nanofiber mats based devices were formed by sputtering Pt to form electrodes using shadow mask technique with the fixed electrode gap distance of 1 mm.

Temperature-dependent electrical properties including current-voltage (I-V) and field-effect transient (FET) measurements were characterized based on the single fiber-based devices at a temperature ranging from 293 to 353 K, while thermopower (S) was measured based on mats by a home-built instrument. The temperature range was varied from 300 to 360 K.

## Results and discussions

### Electrospinning of co nanofibers as sacrificial materials

**Figure 1:**
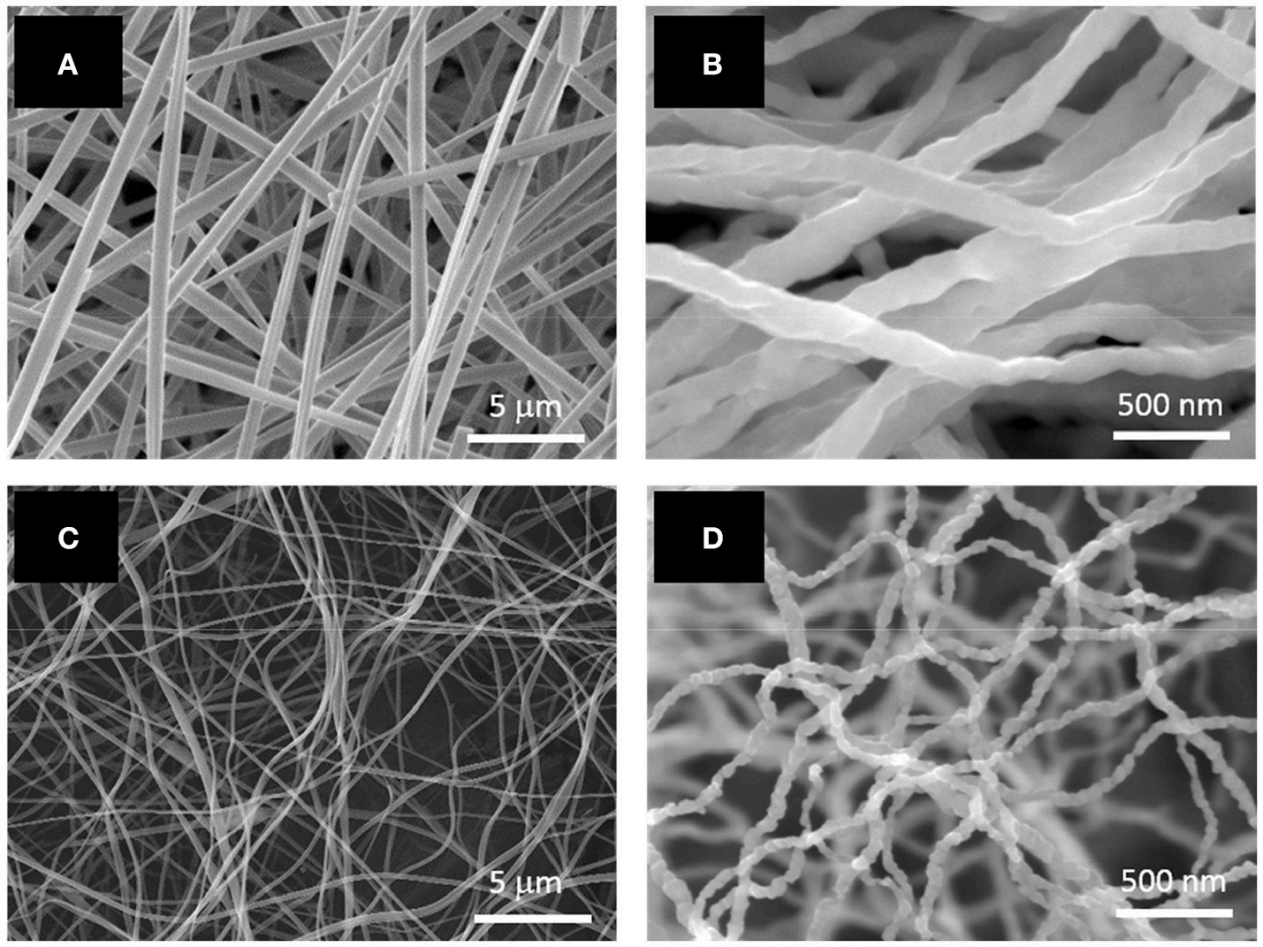
SEM images of electrospun PVP/acetic acid/Co acetate nanofibers with average diameters of **(A)** 394 nm and **(C)** 161 nm; SEM images of Co nanofibers with average diameters of **(B)** 124 nm and **(D)** 52 nm.

Table 1 shows the solution properties of two electrospinning solutions which were investigated. As listed in the table, higher loadings of citric acid and cobalt acetate significantly increased electrical conductivity and viscosity. However, the surface tension of both solutions were almost the same, as expected, since this parameter would mainly depend on the properties of the solvent. Smooth, cylindrical CoAc_2_/citric acid/PVP nanofibers were electrospun from both solutions, as shown in Figures 1A,C. Their average diameters were 519 ± 168 nm and 161 ± 47 nm, respectively. The large standard deviations were likely due to the inhomogeneous electrical bending instability of the fibers as well as the formation of branched fibers, which originated from the nonuniform charge density distribution on the surface of the jet (Reneker and Yarin, 2008). Compared to Figure 1A, the nanofibers in Figure 1C are more curled due to their smaller average diameter. Annealing of the as-spun fibers at 500°C for 5 h in H_2_ environment led to a complete decomposition of the polymer and acid as well as the formation of continuous Co nanofibers (Figures 1B,D). The average diameter of the Co nanofibers was 124 ± 30 nm and 52 ± 12 nm. The smaller Co nanofibers (Figure 1D) seemed to have a rougher surface than the larger ones (Figure 1B). Both nanofibers exhibited a similar volume shrink ratio of approximately 60%.

**Table 1:** Solution properties of electrospinning precursors.

**Solution property**	**Viscosity (cP)**	**Electrical conductivity (mS/cm)**	**Surface tension (dyne/cm)**
Solution 1 (Resulted in smaller Co nanofiber)	82.5	1.0	37
Solution 2 (Resulted in larger Co nanofiber)	130.3	1.1	37

### Fabrication of PbTe hollow nanofibers and their material characteristics

Co was chosen as the sacrificial material for galvanic displacement reaction for the synthesis of PbTe due to its ability to provide an appropriate electrochemical driving force (difference in materials' redox potential) for the metal chalcogenide displacement. Since the redox potential of Co^2+^/Co pair (-0.28 V vs. SHE) is more cathodic than that of the Pb^2+^/Pb (−0.13 V vs. SHE) and ${\text{HTeO}}_{2}^{+}$/Te (0.551 V vs. SHE) pairs, the formation of PbTe from Co was thermodynamically favorable. A suitable dissolution rate of the sacrificial materials was also a key parameter for a successful displacement. Sacrificial materials with a dissolution rate comparable to the deposition rate of the target materials are required to achieve high deposition efficiency and a low sacrificial material residual. Dipping the Co nanofiber mats into the acidic electrolytes containing Pb^2+^ and ${\text{HTeO}}_{2}^{+}$ would lead to the dissolution of Co to Co^2+^ (Equation 1) as well as the formation of PbTe. Here, Co nanofibers served as both the electron source and the template for the PbTe deposition. The deposition would initiate with overpotential deposition of Te nuclei on the surface of the Co nanofibers (Equation 2), due to a more negative standard reduction potential of Co than Te. Pb spontaneously deposited on Te by underpotential deposition (UPD) mechanism, which resulted in the formation of PbTe. PbTe compound is formed instead of Pb and Te due to the negative ΔG of PbTe formation (Equation 3) (Xiao et al., 2006). Galvanic displacement of PbTe from Co can be described in the Equations 1–4:

(1) $$\begin{array}{r} Co\left(s\right)\to Co^{2+}\left(aq\right)+2e^{-}E^{0}=-0.28V\;vs.SHE \end{array}$$

$$\begin{array}{r} HTeO_{2}^{+}\left(aq\right)+4e^{-}+3H^{+}\left(aq\right)\to Te\left(s\right) \end{array}$$

(2) +2H2O              E0=0.551V vs.SHE

(3) $$\begin{array}{r} Pb^{2+}\left(aq\right)+Te\left(s\right)+2e^{-}\to PbTe\left(s\right)\Delta G_{f}^{0}=-69.5\;kJ/mol \end{array}$$

$$\begin{array}{r} Pb^{2+}\left(aq\right)+HTeO_{2}^{+}\left(aq\right)+3Co\left(s\right) \end{array}$$

(4) $$\begin{array}{r} +3H^{+}\left(aq\right)\to PbTe\left(s\right)+3Co^{2+}\left(aq\right)+2H_{2}O \end{array}$$

Control over the dimension and morphology of the PbTe nanofibers was achieved by varying the diameter of Co nanofibers (*i.e*., 52 and 124 nm) and the concentrations of Pb^2+^ (i.e., 10, 50, and 100 mM) in the electrolytes and, while maintaining the concentrations of HNO_3_ and ${\text{HTeO}}_{2}^{+}$ at 0.1 M and 0.1 mM, respectively. Compared to the GDR of PbSeNi, the concentration of HNO_3_ in the PbTe's electrolytes was ten times lower (i.e., 0.1 M) to reduce the dissolution rate of Co, which is much faster than that of Ni (Jung et al., 2012).

**Figure 2:**
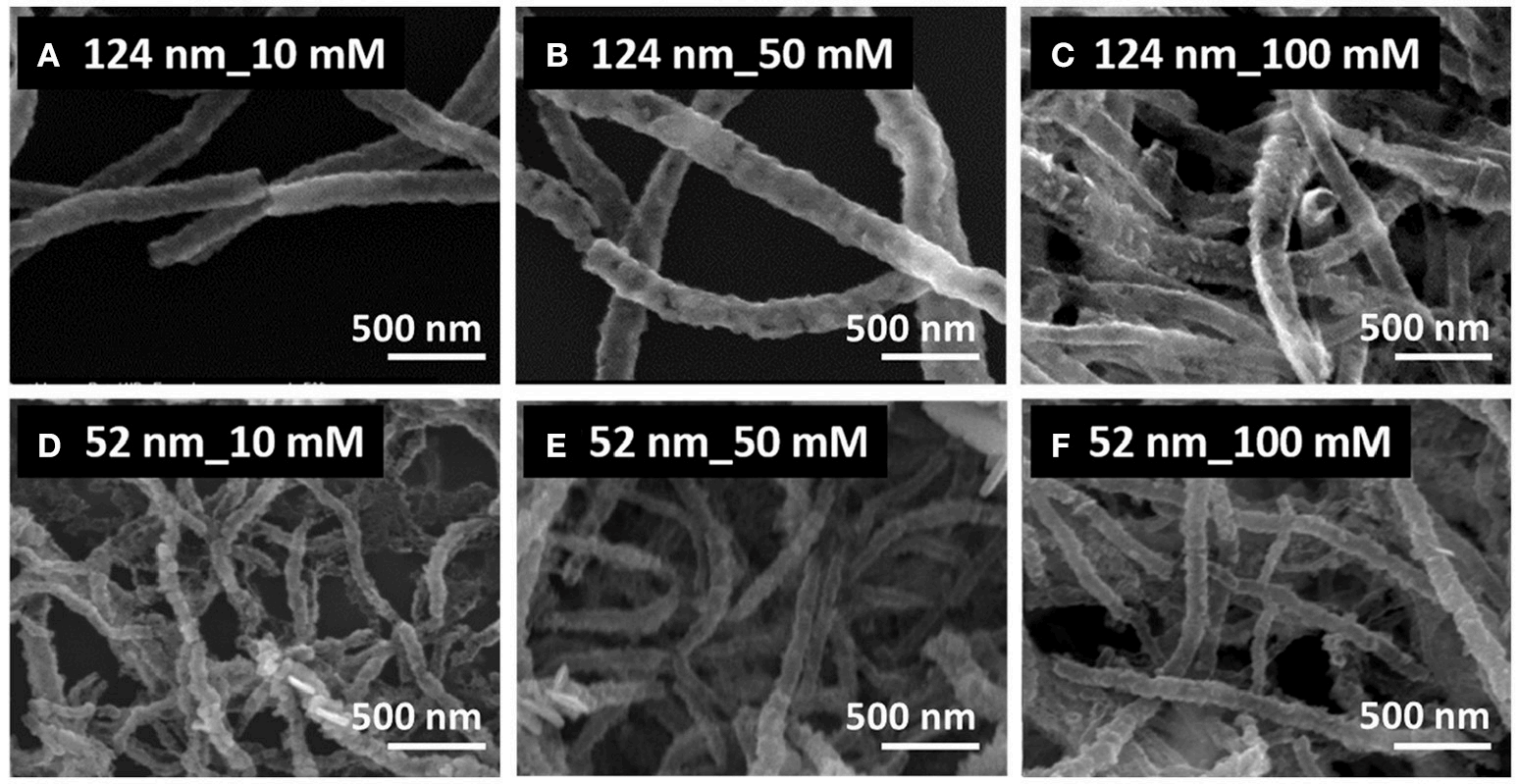
SEM images of synthesized PbTe hollow nanofibers using 124-nm (top row, **A–C**) and 52-nm (bottom row, **D–F**) Co nanofibers as sacrificial materials. The electrolytes contained a fixed concentration of 0.1 mM ${\text{HTeO}}_{2}^{+}$ and 0.1 M HNO_3_ with various concentration of Pb^2+^ of 10 mM (left column, **A,D**), 50 mM (middle column, **B,E**), and 100 mM (right column, **C,F**). All reactions were conducted at room temperature for 30 min.

Figure 2 shows the SEM images of PbTe produced from Co nanofibers with average diameters of 124 nm (top row) and 52 nm (bottom row). The concentration of Pb^2+^ was varied from 10 mM (left column), to 50 mM (middle column), and to 100 mM (right column). All three conditions produced nanofibers, some of them with clear hollow structures (Figures 2A,C). Most of the nanofibers in the top row were consistently larger than that in the bottom row, as the diameter of the sacrificial nanofiber was larger. For the larger PbTe nanofibers (top row of Figure 2), hollow and smooth nanofibers were synthesized in the electrolytes containing low concentrations of Pb^2+^, while nanofibers with rough surfaces were observed from the concentrated electrolytes. For smaller PbTe nanofibers (bottom row of Figure 2), no clear morphology difference was observed with varied Pb^2+^ concentration. A closer look at the low magnification images (Figure S1) showed that these larger fibers were smoother than the smaller fibers. This might be attributed to the smoother surface of the larger sacrificial nanofibers, providing less local nucleation sites for the chalcogenide deposition. The formation of hetero-structures in the smaller PbTe nanofibers might be due to the greater mass transfer limitation resulting from the higher fiber pack density. Opened nanotubes or porous tubes were observed in Figures 2B,C, which might be caused by the incomplete coverage of the deposits on the sacrificial material or the dissolution of the as-deposited materials along the reaction. Fibers merged together with adjacent ones as shown in Figures 2D–F, which might be due to the linking of sacrificial nanofibers as well as the dissolution of the as-deposited PbTe.

The synthesized PbTe nanofiber mats were then sonicated and dispersed in IPA to obtain single nanofiber-based suspension solutions. These solutions were drop-casted on Si/SiO_2_ chips for the EDS characterization. The composition of over six individual fibers were measured and averaged for each condition. The composition of PbTe as a function of Pb^2+^ concentration (Table 2). For the smaller PbTe nanofibers, the Pb content stayed at 42 at.% as the concentration of Pb^2+^ increased from 10 mM to 50 mM, then decreased to 37 at. % at a higher level of Pb^2+^ concentration. However, for the larger PbTe nanofibers, a slight decrease in the Pb content at the low Pb^2+^ concentration region [*i.e*., (Pb^2+^) = 10 mM and 50 mM] was first observed, followed by a slight increase in the Pb content when the concentration of Pb^2+^ was increased to 100 mM. Variations in the ion concentration had minor effects on the fibers' composition because the Pb content maintained at around 42 at.% and only a 7 at. % (from 37 at.% to 44 at.%) change was observed corresponding to a one order of magnitude change in the electrolyte concentration. The insignificant effect of a high Pb^2+^ concentration ([Pb^2+^] ≥ 50 mM) on the composition of PbTe has been suggested in our previous work (Xiao et al., 2006). Large error bars were observed for all the conditions, which might be attributed to the inhomogeneity in the reactions due to the mass transfer limitation. The highest Pb content in the lead chalcogenide nanofibers (i.e., PbSe and PbTe) galvanically displaced from the sacrificial materials that have a similar redox potential (*i.e*., Ni and Co) (Zhang et al., 2014) was around 45 at.%. GDR of PbTe from Co has been studied by Chang et al. (2014). In their system, electrodeposited Co thin film was used as sacrificial anode. The electrolytes consisted of 50 mM to 500 mM Pb^+2^ while fixing ${\text{HTeO}}_{2}^{+}$ concentration at 10 mM. These electrolytes allowed them to study the deposition in the [Pb^2+^]/[${\text{HTeO}}_{2}^{+}$] system ranging from 5 to 50. In our case, however, the deposition occurred in a much higher [Pb^2+^]/[${\text{HTeO}}_{2}^{+}$] range of 100 to 1000 due to the 100-fold lower ${\text{HTeO}}_{2}^{+}$ concentration (*i.e*., 0.1 mM). In Chang's thin film system, Te-rich PbTe films were obtained at [Pb^2+^]/[${\text{HTeO}}_{2}^{+}$] ≤ 20, while Pb-rich PbTe films were synthesized at [Pb^2+^]/[${\text{HTeO}}_{2}^{+}$] ≥ 50 (Chang et al., 2014). However, only Te-rich PbTe were obtained in the nanofiber system, even with a higher [Pb^2+^]/[${\text{HTeO}}_{2}^{+}$] of 1,000. The difference in the composition might be due to the different geometry of the sacrificial material leading to growth of heterostructures in the thin film system, or the difference in the crystal orientation of Co film (hcp) and nanofibers (fcc) that provided different activation energies for the Pb overpotential deposition (OPD) (Oviedo et al., 2006).

**Table 2:** Effect of [Pb^2+^] on the Pb content in the PbTe nanofibers using two different Co nanofibers (i.e., 52 nm and 124 nm).

**[Pb^+2^]**	**Pb content (at. %)**	
	**52 nm Co nanofiber**	**124 nm Co nanofiber**
10	42 ± 4.1	42 ± 6.3
50	42 ± 5.9	38 ± 6.2
100	37 ± 6.8	44 ± 3.3

**Figure 3:**
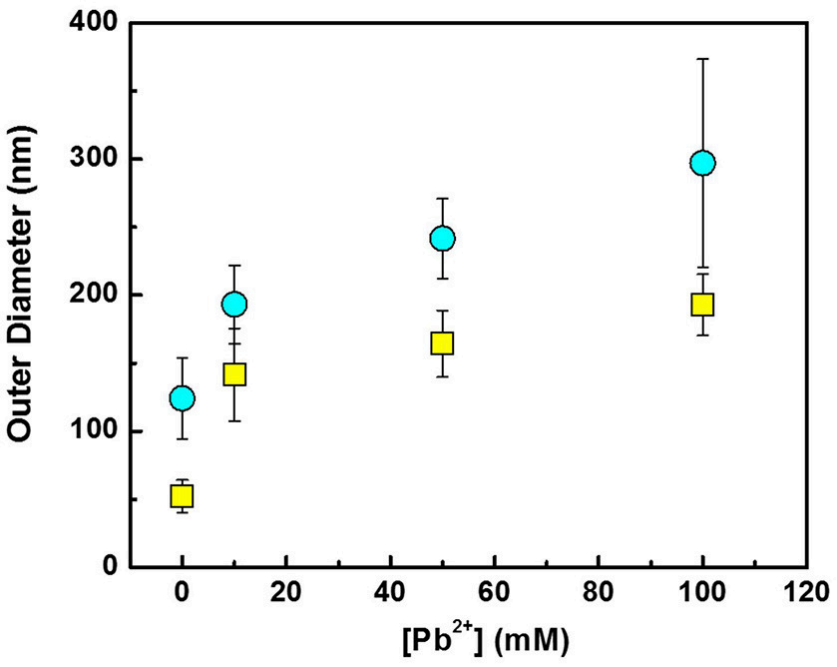
Effect of [Pb^2+^] on the outer diameter of PbTe nanofibers. The outer diameters of Co nanofibers are shown in Figure at [Pb^2+^] = 0. Yellow squares and blue circles indicate PbTe nanofibers from Co nanofibers with the average diameter of 52 and 124 nm, respectively.

A quantitative assessment of the effect of the Pb^2+^ concentrations on the average outer diameter of the Pb_x_Te_y_ nanofibers is shown in Figure 3. Pb^2+^ concentration was kept significantly higher than ${\text{HTeO}}_{4}^{+}$ concentration since the PbTe deposition mechanism follows overpotential deposition (OPD) of Te, followed by underpotential deposition (UPD) of Pb. Nanofibers displaced in the electrolytes with a higher Pb^2+^ concentration exhibited larger outer diameter. As the concentration of Pb^2+^ was increased from 10 mM to 100 mM, the outer diameters increased from 141 to 193 nm for smaller PbTe nanofibers and 193 to 297 nm for larger PbTe nanofibers. Owing to the faster deposition rate, greater outer diameters were expected in the electrolytes containing higher concentrations of Pb^2+^. In addition, the large disparity in the size of the larger fiber diameter may cause the larger error bars in the fiber diameters. The same phenomenon has been observed in previous work (Zhang et al., 2014). The wall thickness of the PbTe nanofibers were estimated assuming the inner diameters of hollow PbTe nanofibers were the same as the outer diameter of Co nanofibers. As the Pb^2+^ concentration was increased from 10 to 50 to 100 mM, the wall thickness increased from 45 to 56 to 70 nm for the smaller nanofibers, and from 35 to 59 to 86 nm for the larger nanofibers. Given that the Bohr radius of PbTe is 46 nm, the quantum confinement effect may occur in the thin nanofibers.

**Figure 4:**
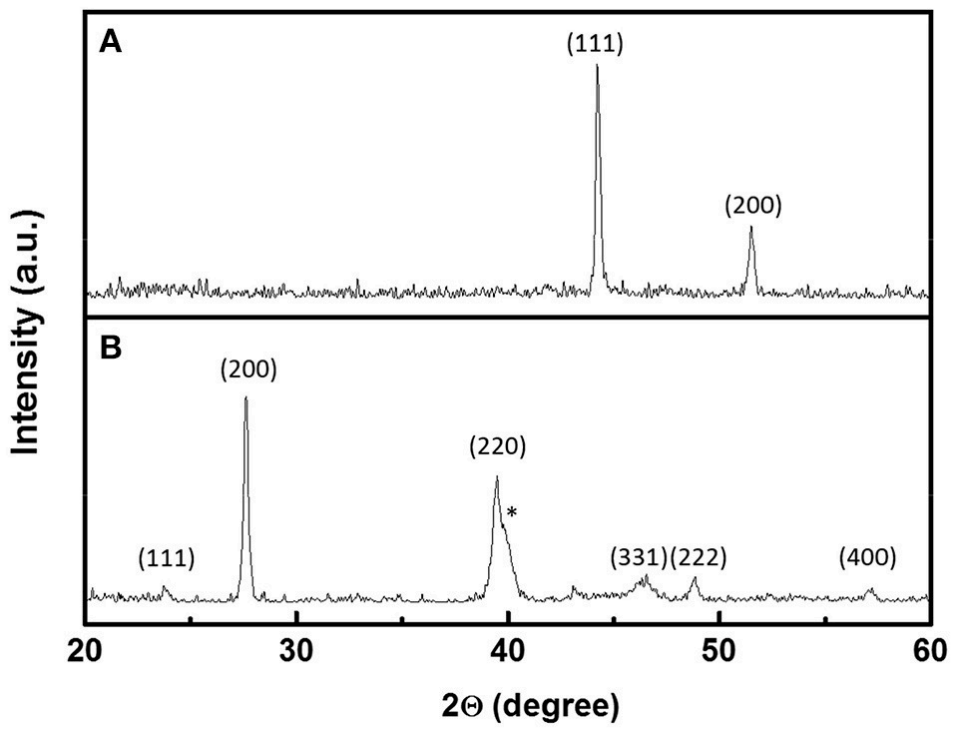
XRD pattern of **(A)** 124-nm Co nanofibers and **(B)** Pb_43_Te_57_ hollow nanofibers from Figure 2A. The peaks with * belong to Pt electrodes.

Figure 4 shows XRD patterns of the Co nanofibers and the Pb_43_Te_57_ nanofibers. Co nanofibers (Figure 4A) had a diffraction pattern of a fcc structure with < 1 1 1> and < 2 0 0> orientations. All the peaks in the PbTe (Figure 4B) matched with PbTe (JCPDS 38-1435) except for the peak that belonged to the Pt electrodes that were sputtered on top of the nanofiber mats, which was marked with an asterisk. The asymmetric peaks at 39.5°C were contributed from both the < 2 2 0> orientation of PbTe as well as the < 1 1 1> plane of the sputtered Pt electrodes. The PbTe nanofibers showed no preferred orientation. The average grain size was calculated to be 60 nm, based on the intensity of the X-ray peak. No peak from elemental Te was observed. Figure S2 shows XRD patterns of Pb_x_Te_y_ nanofibers synthesized from various concentrations of Pb^2+^ and dimensions of Co nanofibers. Similar to the Pb_43_Te_57_ sample shown in Figure 4B, all of the samples showed random crystal orientation. The average grain size of the larger PbTe nanofibers were approximately the same (i.e., around 60 nm), while that of the smaller fibers was about 44 nm. This might be due to the smaller diameter of Co nanofibers, which provided limited sacrificial material source for the deposition of PbTe. Compared to larger PbTe nanofibers (Figure S2A), smaller nanofibers (Figure S2B) possessed a much lower peak intensity, which indicated a lower degree of crystallinity. No Te peak was observed in either of the nanofiber mats.

**Figure 5:**
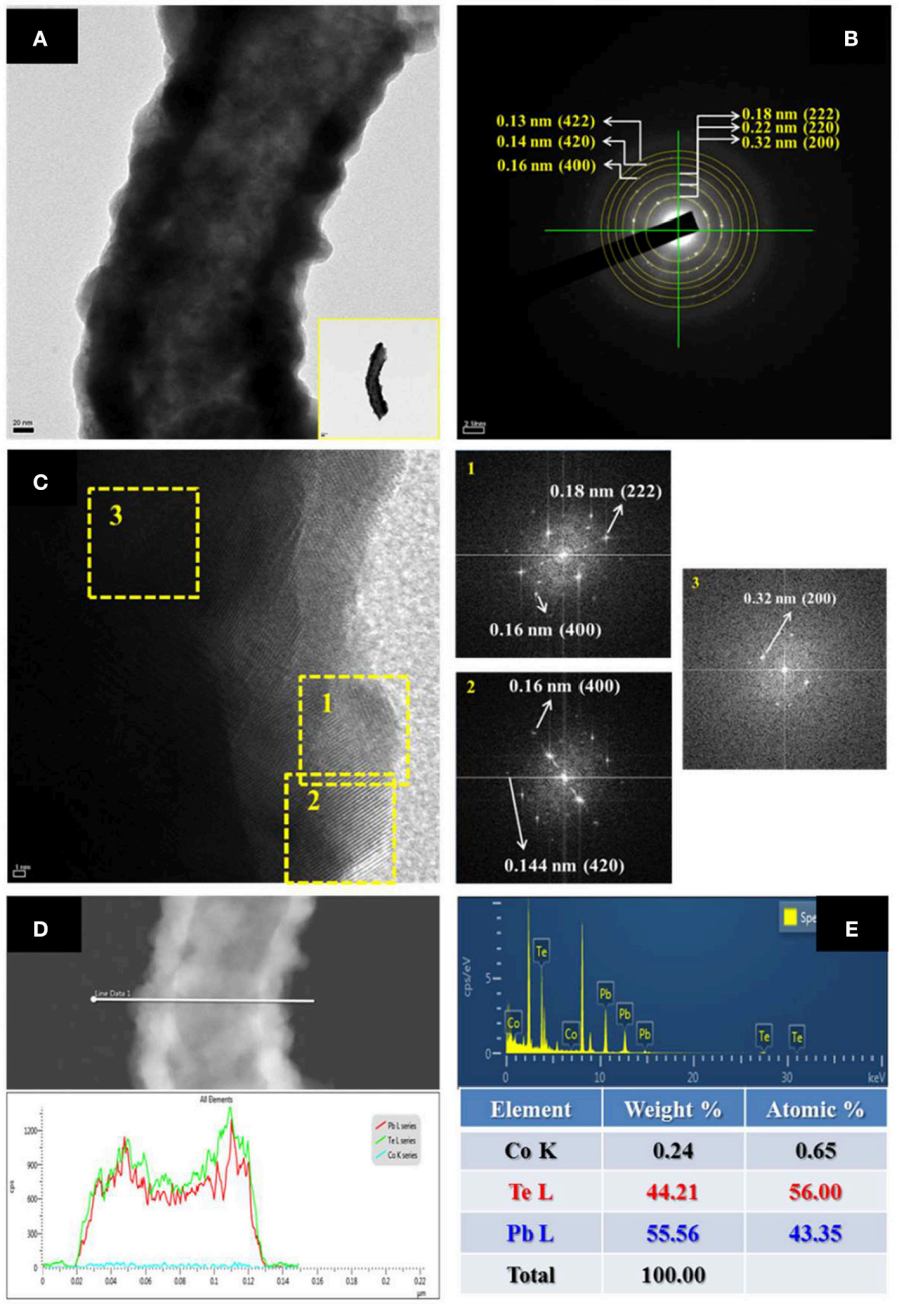
**(A)** HRTEM image (inset: TEM image of low magnification); **(B)** SAED pattern; **(C)** FTT images; **(D)** Line-scan EDS; and **(E)** EDS analysis of Pb_43_Te_56_ hollow nanofibers. The electrolyte consisted of 50 mM Pb^2+^, 0.1 mM ${\text{HTeO}}_{2}^{+}$, and 0.1 M HNO_3_ at room temperature.

High-resolution transmission electron microscopy (HR-TEM) with EDS and SAED were utilized to characterize the morphology, composition, and crystal structure of nanofibers (Figure 5). As shown in the figure, the as-prepared nanofiber showed a nodular and hollow structure (Figure 5A) with Pb content of 43 at. %. The line-scan EDS (Figure 5D) analysis showed that the composition of nanofibers was uniform throughout the fiber. The fast Fourier transform (FFT)-converted SAED patterns indicated that the diffraction pattern came from (222), (220), and (200) orientation of PbTe.

**Figure 6:**
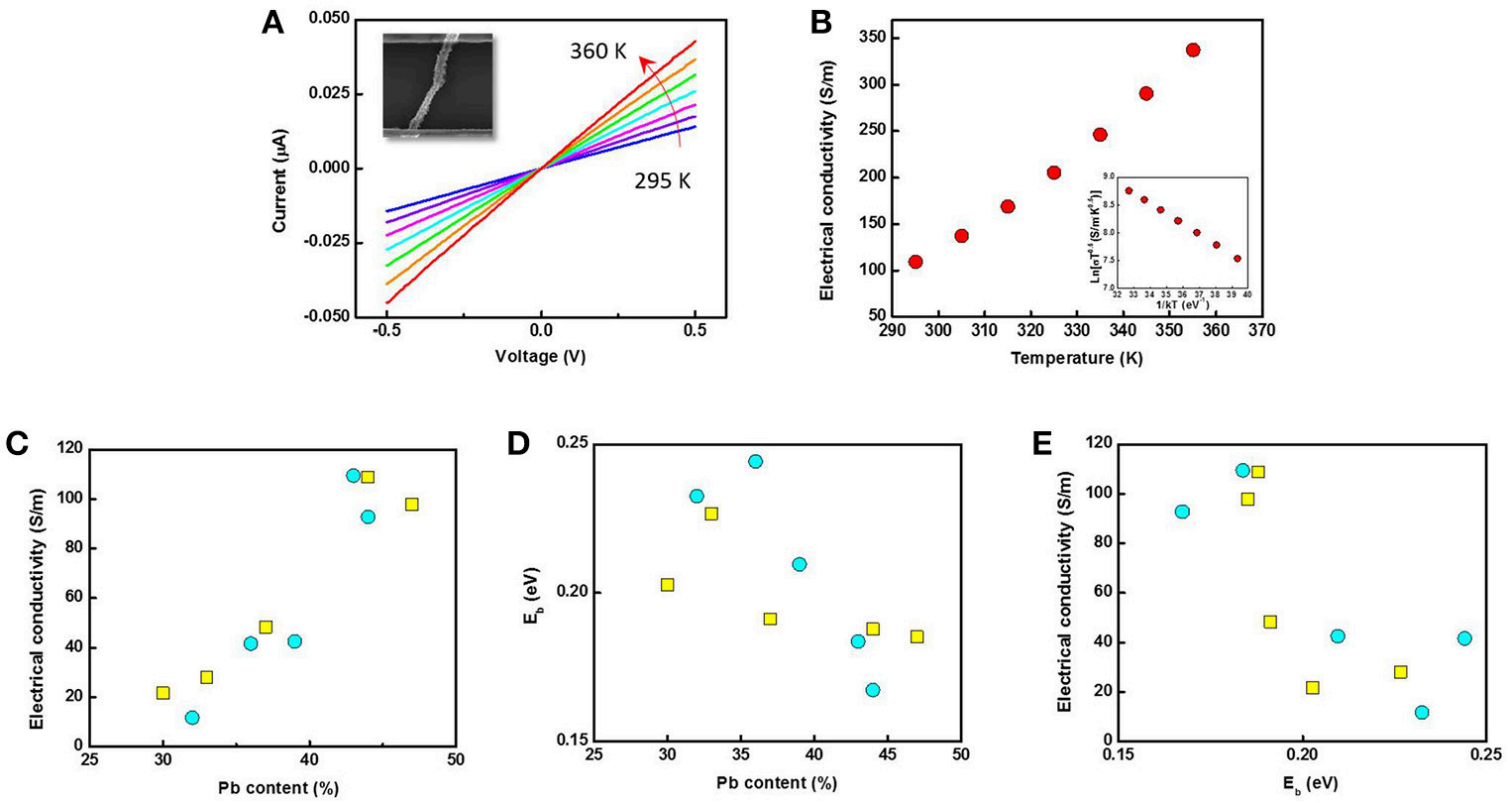
Temperature-dependent **(A)** I-V characterization and **(B)** electrical conductivity of a single Pb_43_Te_57_ nanofiber (inset: Ln(σT^0.5^) as a function of 1/kT). **(C)** Electrical conductivity and **(D)** energy barrier height E_b_ as a function of Pb content. **(E)** Electrical conductivity of PbTe single nanofibers as functions of energy barrier height, E_b_. In Figure c, d and e, yellow squares and blue circles indicate PbTe nanofibers from Co nanofibers with the average diameter of 52 nm and 124 nm, respectively.

Temperature-dependent I-V characterizations were carried out based on single nanofibers where the temperature was varied from 295 to 360 K (Figure 6A). In this temperature range, all the samples show linear I-V characteristics which indicate that the contact between nanofiber and electrodes were ohmic. Figure 6B shows σ as a function of temperature with the insert plot of ln (σT^0.5^) as a function of 1/kT. Here, k is the Boltzmann's constant. The electrical conductivity of single nanofiber was estimated from conductance of nanofiber, the estimated cross sectional area and length of hollow nanofiber. The sample showed an increase in σ with increased temperature, suggesting that the sample is semiconductor which has an energy barrier (E_b_). By fitting the inserted figure in Figure 6B, E_b_ was calculated (Scheele et al., 2011). Figures 6C,D show σ and E_b_ as a function of their Pb content. The electrical conductivity increased by almost an order of magnitude when the Pb content increased by 66% in both sizes of nanofibers, whereas the energy barrier height decreased with the Pb content. The lower electrical conductivity in the lower Pb-content nanofibers might be due to the higher amount of Te, which may have created a larger energy barrier along the sample due to its amorphous phase. A more sensitive effect of Pb content on E_b_ was found in the larger PbTe nanofibers, shown by its steeper slope than the smaller PbTe fibers. This might be due to the larger grain size in the larger nanofibers, to which the electrical conductivity is also proportional (Seto, 1975). As shown in Figure 6E, the electrical conductivity was plotted as a function of E_b_, which showed a monotonically decreasing trend. σ increased one order of magnitude by decreasing in E_b_ of 0.1 eV. A similar trend has been predicted by Faleev in a Pb nanoinclusion embedded PbTe bulk material due to the energy filtering effect (Faleev and Leonard, 2008). However, a much more significant dependence of electrical conductivity on E_b_ was observed in our case, since only a maximum of a 2-fold reduction was suggested in Faleev's model. This may be due to the improved charge carrier movement via non-planar radial transport as compared to 3-D planar transport in bulk materials. The effect of outer diameter of nanofibers on the electrical conductivity and E_b_ is shown in Figure S3. As no clear trend was observed, it could be concluded that the electrical conductivity and energy barrier height of PbTe nanofibers mainly depended on the composition rather than the dimension.

**Figure 7:**
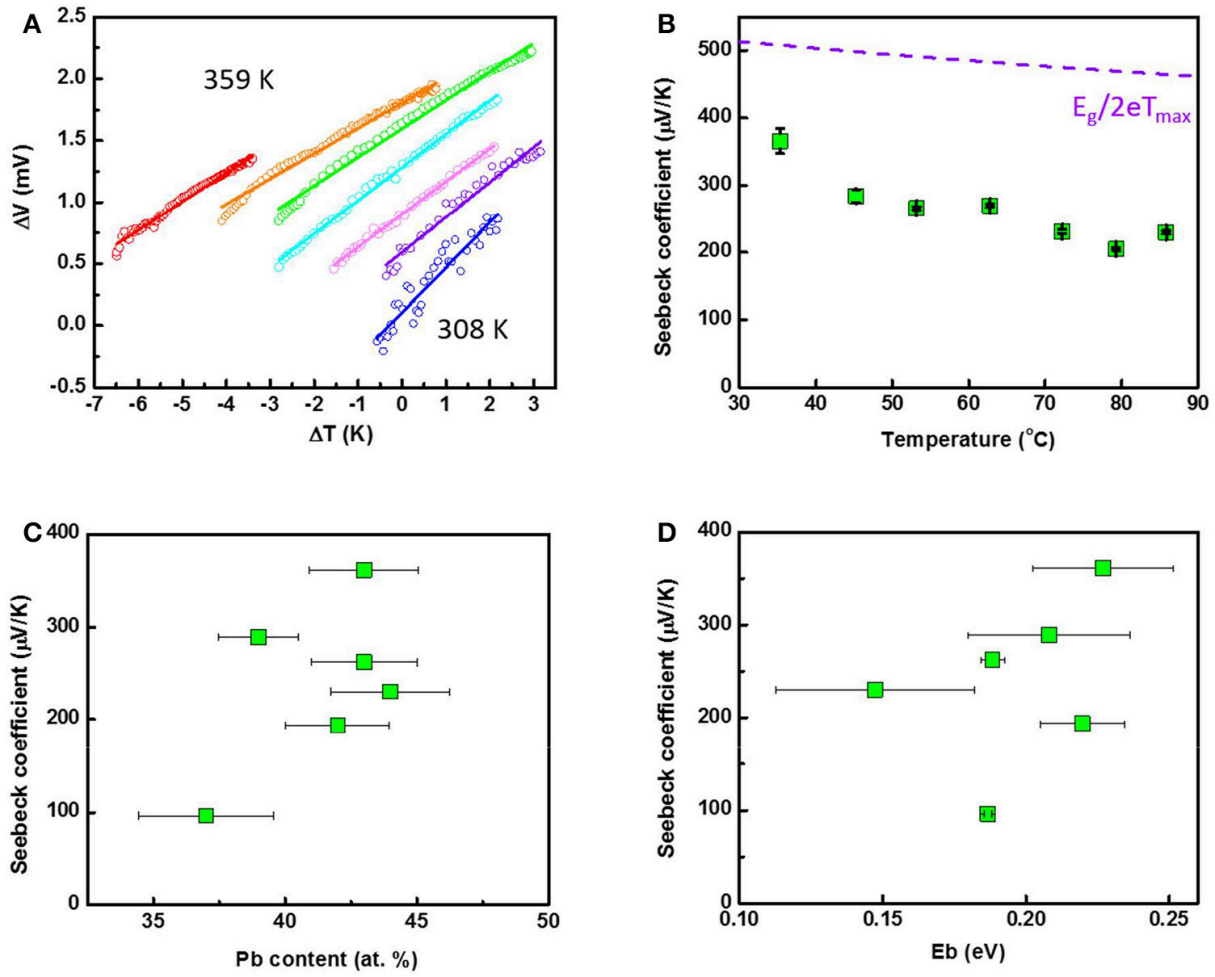
Temperature-dependent **(A)** ΔV-ΔT characterization and **(B)** Seebeck coefficient of Pb_43_Te_57_ nanofiber mat. The maximum Seebeck coefficient S_max_ = E_g_/2eT_max_ was predicted and plotted as the purple dotted line. Seebeck coefficient of PbTe as functions of **(C)** Pb content and **(D)** energy barrier height, E_b_.

Figure 7A shows the temperature dependent ΔV-ΔT of the Pb_43_Te_57_ nanofiber mat where the temperature was varied from 300 K to 360 K. Thermopower (Seebeck Coefficient, S) were determined from the slope of ΔV-ΔT. The temperature-dependent Seebeck coefficients were plotted in Figure 7B. Positive thermopower (S) indicated that the samples are p-type semiconductors. Additionally, lower S with increasing temperature indicating that the sample are closed to intrinsic semiconductor (i.e., low carrier concentration). The highest S of 366 μV/K was observed at 308 K, which was higher than bulk counterpart (Abrams and Tauber, 1969). For a bulk, the maximum Seebeck coefficient (S_max_) is a function of effective band gap energy (i.e., S_max_ = E_g_/2eT_max_) (Goldsmid and Sharp, 1999). Therefore, S_max_ of PbTe was predicted as shown in the dashed line in Figure 7B, knowing that the band gap of PbTe bulk material would follow the equation E_g_ = 0.0004T + 0.19. Because no optimum Seebeck coefficient was achieved in our temperature-dependent study, no comparison was made between the theoretical prediction and our experimental data.

Temperature-dependent Seebeck coefficient of Pb_x_Te_y_ nanofiber mats with various x are shown in Figure S4. Smaller PbTe (i.e., Pb_43_Te_57_, Pb_42_Te_58_, and Pb_37_Te_63_) nanofibers that were reacted from 52-nm Co nanofiber mats are shown in Figure S4a, while larger PbTe (i.e., Pb_43_Te_57_, Pb_39_Te_61_, and Pb_44_Te_56_) nanofibers that were reacted from 124-nm Co nanofiber mats (Figure S4B). The thermopowers, S, were positive in all samples, indicating that they are p-type semiconductors. It is known that the thermopowere consists of partial thermopowers that are contributed by holes and electrons (Goldsmid and Sharp, 1999). Thus, thermopower of instinsic or near-instinsic semiconductor increases with increasing temperature due to greater carrier mobility at high temperature whereas thermopower of highly doped/degraded semiconductor initially increases with temperature upto onset temperature followed by decreasing thereafter (Abrams and Tauber, 1969). The enhancement of the thermopower with temperature is caused by change of Fermi level from the band edge. After onset temperature, the decrease in the thermopower is due to biopolar effects (Dow et al., 2009).

In our experiments, higher Pb content (i.e., Pb_43_Te_57_ and Pb_42_Te_58_) smaller nanofibers show the thermopower increased with temperature (Figure S4A). This was a typical behavior of near-intrinsic semiconductors. However, for fibers with relatively lower Pb content (i.e., Pb_37_Te_63_), the thermopower has an onset temperature at 320 K. Unfortunately, the almost plateauing tendancy of the thermopower cannot be explained at this stage. For the larger PbTe nanofibers (Figure S4B), the thermopower decreased with temperature for all samples. The difference behaviors require further investigation.

Figure 7C shows the thermopower as a function of the Pb content. As expected, higher thermopower were observed when the composition in near stoichiometric due to lower carrier concentration. The Seebeck coefficient describes the ability of the carrier transport from the Fermi level to the conduction band corresponding to a temperature difference. Therefore, a lower Seebeck coefficient is expected in a Te-rich PbTe nanofibers since its Fermi level has been pushed into the conduction band.

Figure 7D shows the thermopower as a function of E_b_. Boundaries and interfaces can acted as an energy filter for the charge carriers, which would enable the transport only from the high charge carriers: a phenomenon known as the energy filtering effect. By doing so, the average energy of the charge carriers increases, which resulted in enhancement of the thermopower. As shown in Figure 7D, the thermopowers increased with the E_b_ of the nanofibers, which was consistent with the prediction (Faleev and Leonard, 2008). The thermopower was also plotted against the average grain size of the nanofiber mats (Figure S5). The smaller PbTe nanofibers had an average grain size of around 45 nm, while the larger PbTe fibers' grain size was approximately 60 nm.

## Conclusions

PbTe nanofiber mats were fabricating using electrospun cobalt nanofibers as the sacrificial materials. Control over the dimension and morphology of the nanofibers were achieved by applying sacrificial material with various diameters and tuning the concentration of Pb^2+^ in the electrolytes during galvanic displacement reaction. Hollow PbTe nanofibers were synthesized in all the conditions. The fibers with larger outer diameter were obtained from thicker Co nanofibers. For the larger PbTe nanofibers, hollow and smooth surfaces were achieved using electrolytes containing low concentrations of Pb^2+^, whereas rough surfaces were observed from using concentrated electrolytes. The formation of rough surface in the latter case may be due to the faster reaction rate. On the other hand, for the smaller PbTe nanofibers, no clear differences in morphology were observed with various [Pb^2+^]. The smaller PbTe nanofibers were rougher than the larger PbTe nanofibers, which might be due to the rougher surface of small Co nanofibers. The Pb^2+^ concentration had negligible effects on the fibers' composition in the studied range. No residue of Co was observed in the fibers after the galvanic displacement reactions, which indicated a complete reaction. The outer diameter of PbTe nanofibers increased with the Pb^2+^ concentration. XRD analysis showed that all synthesized PbTe samples were polycrystalline in nature.

The temperature-dependent I-V characterization was conducted based on single PbTe nanofibers. The electrical conductivity decreased as the Pb content in the nanofibers decreased. It could be suggested that the excess Te created barriers in the nanofibers, increasing the barrier height while decreasing the electrical conductivity.

## Author contributions

MZ, JK, and MN conducted the experiments. SK conducted TEM analysis. S-DP, YC, JL, and NM provided funding and inputs to the manuscript.

### Conflict of interest statement

The authors declare that the research was conducted in the absence of any commercial or financial relationships that could be construed as a potential conflict of interest.
