# Supplementary material for: Synthesis and Thermoelectric Characterization of Lead Telluride Hollow Nanofibers
Source: Front Chem. 2018 Sep 24;6:436. doi: 10.3389/fchem.2018.00436 (PMC6165867; doi:10.3389/fchem.2018.00436)
Supplement: Supplementary file 1 [file Table_1.DOCX]

**Supporting Information**

**Synthesis and Thermoelectric Characterization of Lead Telluride Hollow Nanofibers**

**Miluo Zhang^1^, Su-Dong Park^2^, Jiwon Kim^3^, Michael Nalbandian^4^, Seil Kim^3,5^, Yongho Choa^5^, Jaehong Lim^3^, and Nosang V. Myung^1,^***

^1^Department of Chemical and Environmental Engineering and Winston Chung Global Energy Center, University of California-Riverside, Riverside, CA, USA

^2^Advanced Materials and Application Research Division, Korea Electrotechnology Research Institute, Changwon, South Korea

^3^Electrochemistry Department, Korea Institute of Materials Science, Changwon 641-831, Korea

^4^Department of Civil Engineering and Construction Management, California Baptist University, Riverside, CA, USA

^5^Department of Fusion Chemical Engineering, Hanyang University, Ansan, South Korea

**
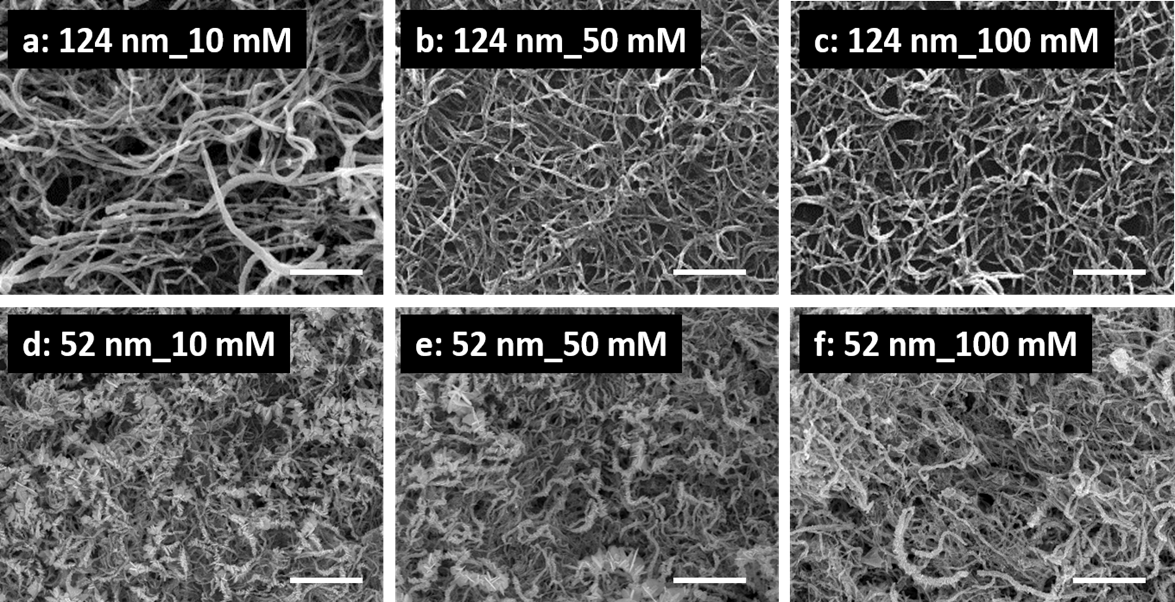
**

**Figure S1**. Low magnification SEM images of synthesized Pb_x_Te_y_ hollow nanofibers using 124 nm (top row) and 52 nm (bottom row) Co nanofibers as sacrificial materials. The electrolytes contain a fixed concentration of 0.1 mM HTeO_2_^+^ and 0.1 M HNO_3_ with various concentration of Pb^2+^ of 10 mM (left column), 50 mM (middle column), and 100 mM (right column). The reactions were conducted at room temperature for 30 min. The scale bar represents 1 micron.


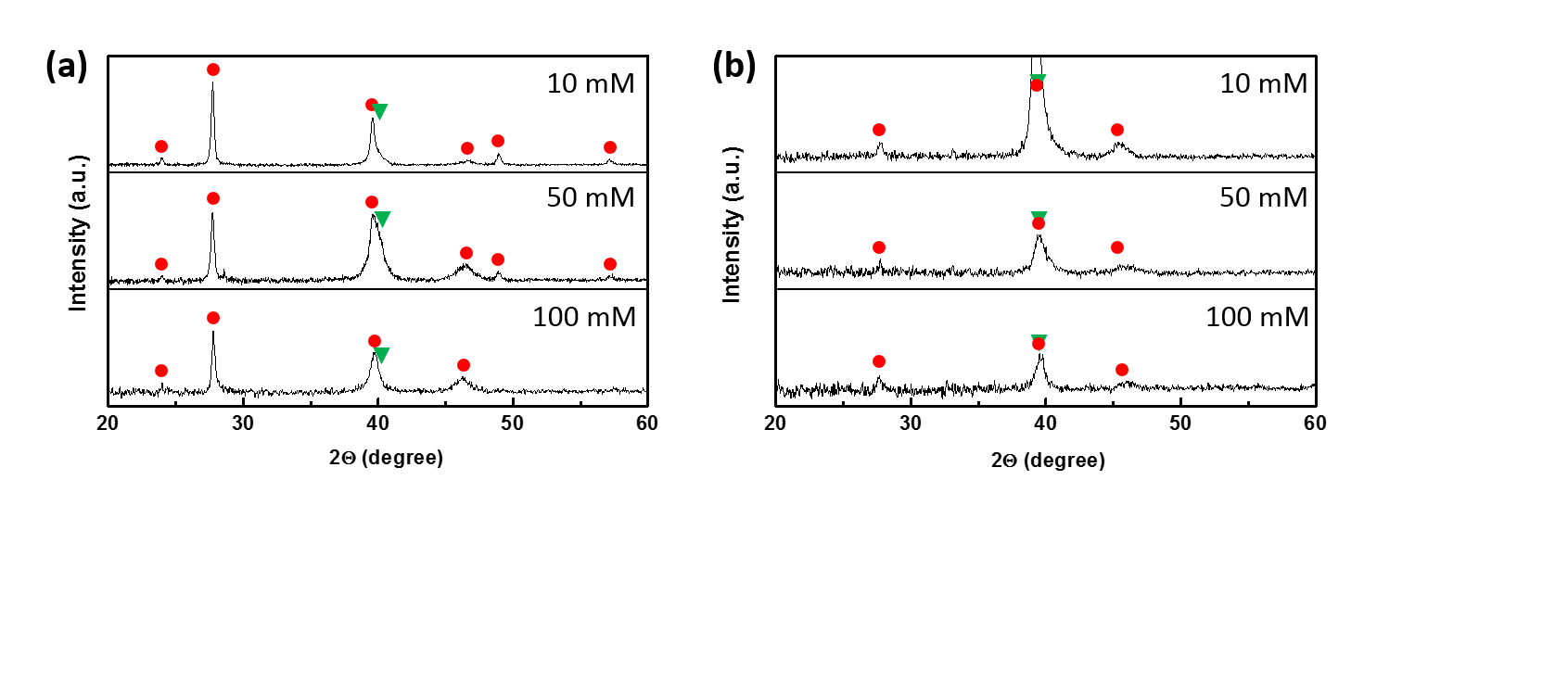


**Figure S2**. XRD pattern of PbTe hollow nanofibers using (a) 124 nm and (b) 52 nm Co nanofibers as sacrificial materials. The electrolytes contain a fixed concentration of 0.1 mM HTeO_2_^+^ and 0.1 M HNO_3_ with various concentration of Pb^+^ of 10 Mm, 50 mM, and 100 mM, as labelled. The peaks with red dots and green triangles belong to PbTe and Pt, respectively.


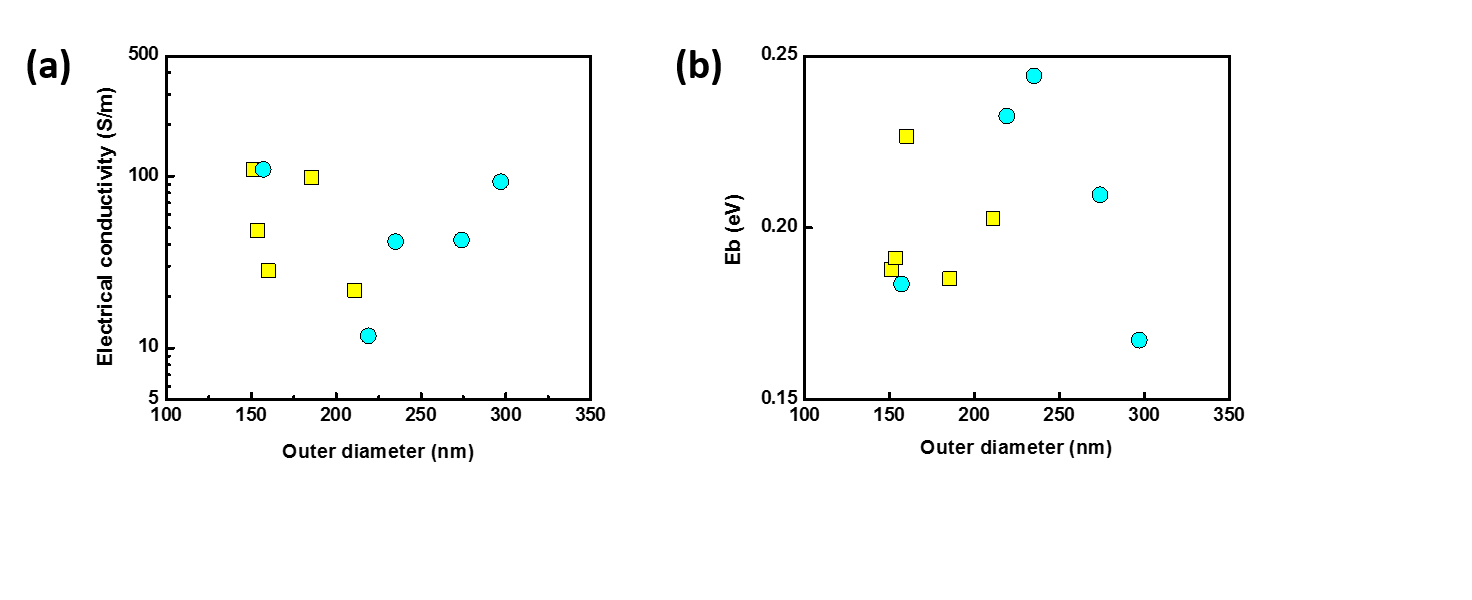


**Figure S3**. (a) Electrical conductivity and (b) energy barrier height E_b_ of PbTe single nanofibers as a function of outer diameter. Yellow squares and blue circles indicate Pb_x_Te_y_ from 52 nm and 124 nm Co nanofibers, respectively.


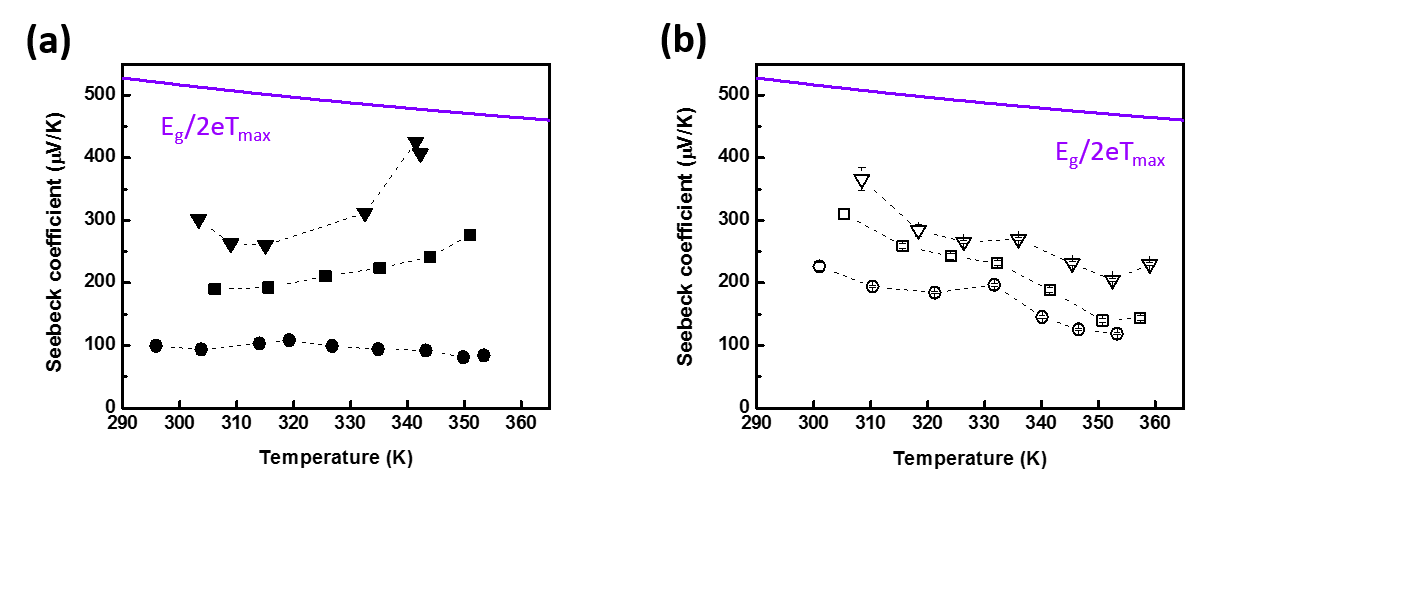


**Figure S4**. (a) Temperature dependent Seebeck coefficient of Pb_43_Te_57_ (solid triangles), Pb_42_Te_58_ (solid squares), and Pb_37_Te_63_ (solid circles) nanofiber mats reacted from 52 nm Co nanofiber mats. (b) Temperature dependent Seebeck coefficient of Pb_43_Te_57_ (open triangles), Pb_39_Te_61_ (open squares), and Pb_44_Te_56_ (open circles) nanofiber mats reacted from 124 nm Co nanofiber mats. The electrolytes contain a fixed concentration of 0.1 mM HTeO_2_^+^ and 0.1 M HNO_3_ with various concentration of Pb^+^ of 10 mM (triangle), 50 mM (square), and 100 mM (circle). The maximum Seebeck coefficient S_max_=E_g_/2eT_max_ is predicted and plotted in the purple lines.


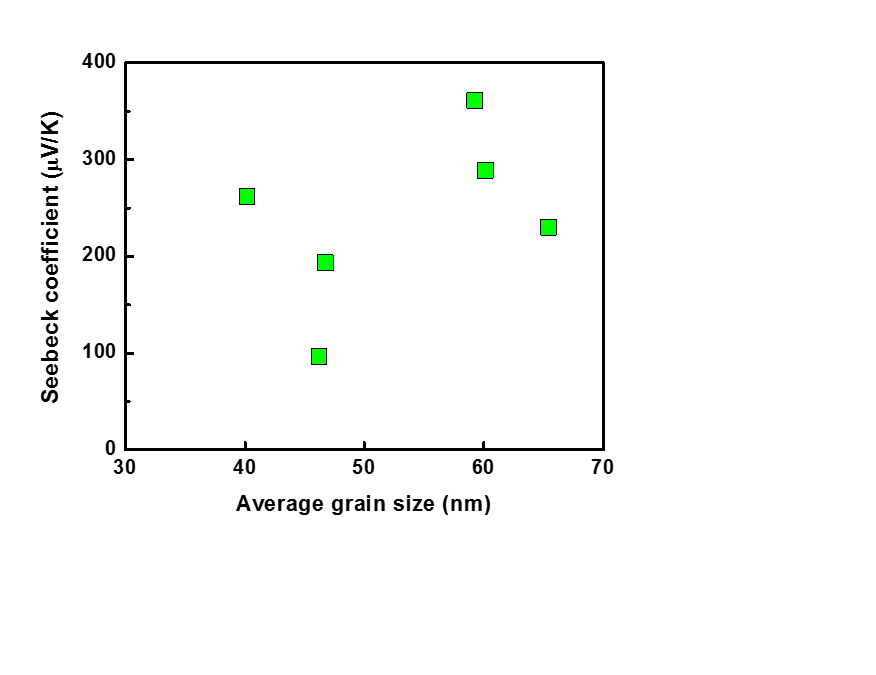


**Figure S5**. Seebeck coefficient of PbTe nanofiber mats as a function of average grain size.
